# Copper Trafficking in Plants and Its Implication on Cell Wall Dynamics

**DOI:** 10.3389/fpls.2016.00601

**Published:** 2016-05-06

**Authors:** Bruno Printz, Stanley Lutts, Jean-Francois Hausman, Kjell Sergeant

**Affiliations:** ^1^Environmental Research and Innovation Department, Luxembourg Institute of Science and TechnologyEsch-sur-Alzette, Luxembourg; ^2^Groupe de Recherche en Physiologie Végétale, Earth and Life Institute Agronomy, Université catholique de LouvainLouvain-la-Neuve, Belgium

**Keywords:** copper, transport, plant, cell wall

## Abstract

In plants, copper (Cu) acts as essential cofactor of numerous proteins. While the definitive number of these so-called cuproproteins is unknown, they perform central functions in plant cells. As micronutrient, a minimal amount of Cu is needed to ensure cellular functions. However, Cu excess may exert in contrast detrimental effects on plant primary production and even survival. Therefore it is essential for a plant to have a strictly controlled Cu homeostasis, an equilibrium that is both tissue and developmentally influenced. In the current review an overview is presented on the different stages of Cu transport from the soil into the plant and throughout the different plant tissues. Special emphasis is on the Cu-dependent responses mediated by the SPL7 transcription factor, and the crosstalk between this transcriptional regulation and microRNA-mediated suppression of translation of seemingly non-essential cuproproteins. Since Cu is an essential player in electron transport, we also review the recent insights into the molecular mechanisms controlling chloroplastic and mitochondrial Cu transport and homeostasis. We finally highlight the involvement of numerous Cu-proteins and Cu-dependent activities in the properties of one of the major Cu-accumulation sites in plants: the cell wall.

## Plant Mineral Homeostasis

Plant metabolism requires not only light but also water and specific elements to allow growth and development. Throughout evolution, plants have developed specialized structures and functions allowing an efficient access to these elements and their balanced distribution among the organism. Seventeen elements are considered to be essential for all vascular plants, grouped in two main classes depending whether they are essential at low concentrations (usually below 100 mg/kg DW), the micronutrients, or if the concentrations needed are higher (more than 1000 mg/kg DW), the macronutrients. A panel of specific, but relatively well-conserved transporters, channels, and pumps has evolved, allowing plants to adjust their mineral uptake, translocation, and distribution capacities in response to environmental fluctuations. Such mineral transport involves different mechanisms relying on proteins, including members of the zinc-regulated transporters (ZRT), iron-regulated transporter (IRT)-like protein (ZIP) family, heavy metal ATPases (HMA) proteins of the P_1B_-type ATPase family and members of the cation diffusion facilitator (CDF) family or yellow stripe like (YSL) transporters.

### Occurrence of Cu in the Environment

Copper has a natural abundance of 60 mg/kg in the Earth’s crust. In Europe, average ambient background Cu concentrations in soils vary between 11.4 and 17 mg Cu/kg (Alloway, 2013). Recommended Cu intake for humans usually ranges from 0.2 mg/day/person for infants to 1.3 mg/day/person for females under lactation (Trumbo et al., 2001). Although human Cu needs are low, Cu deficiency can occur because of its low concentration in edible plant tissues, especially when plants are grown on calcareous or alkaline soils in arid and semi-arid environments (White and Broadley, 2009). Enhancing the phytoavailability of Cu by modulating the molecular and physiological processes involved in the uptake, distribution and accumulation of Cu has consequently been integrated in biofortification programs (White and Broadley, 2009). In contrast, the use of Cu as fungicide has been generalized in agriculture since the late 19th century (Alloway, 2013). In viticulture, Cu-based fungicides are used at typical applications of 2–4 kg Cu/ha/year, leading to soil concentrations of Cu that may reach values higher than 3000 mg Cu/kg of soil, thereby surpassing the concentration range tolerable for most cultivated crops and thus preventing their growth (Alloway, 2013).

### Role of Cu in Plant Biology

The rise of photosynthetic organisms on Earth has driven a progressive accumulation of oxygen in the environment. This oxidative atmosphere led to a decreased solubility of iron by the formation of iron oxides and to the progressive liberation of soluble Cu(II) from insoluble Cu sulfide salts (Burkhead et al., 2009). Since then, iron in biological molecules has been progressively substituted by Cu which is able to perform similar functions. This explains why many Cu-proteins have a functional counterpart that uses Fe as cofactor and why growth on a substrate with a toxic Cu level is commonly linked to a decreased Fe-content in roots and leaves (Pätsikkä et al., 2002; Burkhead et al., 2009; Festa and Thiele, 2011). Consequently, plant phenotypes associated with Cu toxicity share similarities with those related to Fe-deficiency, such as the presence of leaf chlorosis, decreased leaf chlorophyll content and enhanced oxidative stress (Pätsikkä et al., 2002). In contrast, copper deficient plants develop chlorotic symptoms that appear first at the tip of the young leaves prior forming necrotic lesions. Plants grown under Cu deficiency also show impairment in the photosynthetic transport chain and a reduction in non-photochemical quenching, which is consistent with a lack of plastocyanin (PC) function (Abdel-Ghany and Pilon, 2008).

Under physiological conditions, the transition metal Cu is found in the two common forms, the reduced Cu(I) state and the oxidized Cu(II) state. Depending on this state, Cu can bind different substrates. In its reduced form Cu(I) preferentially binds sulfur-containing compounds having a thiol or a thioether group, whereas the oxidized form Cu(II) coordinates mainly with oxygen or imidazole nitrogen groups (Cohu and Pilon, 2010). This dual chemistry of Cu allows it to interact with a wide range of molecules, in particular proteins, to drive biochemical reactions or stabilize structural features (Festa and Thiele, 2011). However, being redox active free Cu can directly lead to the generation of reactive oxygen species (ROS) through Fenton chemistry thereby causing damage to proteins, DNA and other biomolecules (Hänsch and Mendel, 2009).

In living organisms, the main functions of Cu are the transport of electrons in mitochondria and chloroplasts (the most abundant Cu protein is plastocyanin, a photosynthesis-related protein involved in the transfer of electrons from cytochrome f to P700+), the control of the cellular redox state (a major Cu-binding protein is the Cu/Zn superoxide dismutase) but also the remodeling of the cell wall (Cohu and Pilon, 2010).

### Copper Homeostasis Is Mediated by SPL7 Regulation

Most advances in understanding the regulation of Cu homeostasis have been obtained from studies wherein plants were subjected to Cu deficiency. In photosynthetic organisms, Cu deprivation induces a cascade of reactions aimed at a strict economy in Cu-use in order to optimize its delivery to the most essential cellular processes.

**FIGURE 1:**
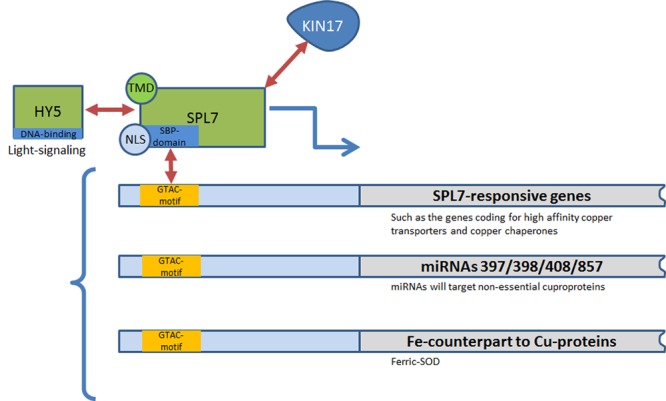
**SPL7-regulation network and existing interactions in plants.** HY5, BZip transcription factor ELONGATED HYPOCOTYL 5; KIN17, Kin17 DNA and RNA Binding Protein; NLS, nuclear localization signal; SBP-domain, SQUAMOSA promoter binding proteins-domain; SOD, superoxide dismutase; SPL7, SQUAMOSA promoter-binding protein-like 7 transcription factor; TMD, transmembrane domain.

Copper homeostasis is mainly regulated by a central Cu homeostatic machinery which involves the Cu-responsive transcription factor SPL7 (for SQUAMOSA promoter-binding protein-like), belonging to the SQUAMOSA promoter binding proteins (SBPs) family. SPL7 is a functional homolog of the Cu response regulator 1 (CRR1), being a similar SBP-domain transcription factor involved in Cu-signaling in *Chlamydomonas reinhardtii.* SBP-domains are highly conserved DNA-binding domains able to recognize the site TNCGTACAA and more specifically the GTAC core sequence (**Figure 1**). The SBP-domain contains two zinc finger-like structures (ZF1 and ZF2), with each Zn ion coordinated by four Cys or His residues (Yamasaki et al., 2004; Kropat et al., 2005; Sommer et al., 2010). Zn(II)-binding to ZF1 is essential for a proper folding of the SBP domain whereas Zn(II)-binding to ZF2 ensures a high-affinity for DNA binding (Sommer et al., 2010). In *C. reinhardtii* high Cu concentrations abolish the high-affinity DNA binding of SBP by the specific interaction with two His residues (Sommer et al., 2010). In plants, evidences of direct interaction between SPL7 and Cu are still lacking. However, it was hypothesized that, in the presence of sufficient Cu, SPL7 may bind Cu by interacting with specific Cu-complexes, thereby resulting in the inability of SPL7 to bind the GTAC motif in the promotor of target genes (Garcia-Molina et al., 2014b). This important aspect of SPL7-regulation of Cu-homeostasis requires further study.

SPL7 was shown to be constitutively expressed in plants – although mainly in roots – independently of soil Cu availability. Consequently, part of its regulation may occur at the post-transcriptional level (Yamasaki et al., 2009; Garcia-Molina et al., 2014a). Garcia-Molina et al. (2014a) identified KIN17, a nuclear protein able to interact with SPL7 *in vivo* that may be involved in such a regulation (**Figure 1**). By studying the development of the plant phenotype of the *Arabidopsis* double mutant *kin17-1 spl7-2* under different Cu-conditions, a concerted action of KIN17 and SPL7 appears essential to alleviate growth defects and oxidative stress in case of Cu deprivation (Garcia-Molina et al., 2014a). Immunolocalization of SPL7 and an ER-marker further revealed the possible dual subcellular localization of SPL7 in both the nucleus and the ER (Garcia-Molina et al., 2014b). It has been hypothesized that Cu-deficiency induces ER stress, thereby promoting activation of SPL7. In addition, SPL7 may homodimerize outside the nucleus, preventing its entry in the nuclear pore due to a larger size or the masking of the nuclear localization signal (NLS) necessary for nuclear import, as a negative feedback mechanism (**Figure 1**; Birkenbihl et al., 2005; Garcia-Molina et al., 2014b).

SPL7 has furthermore been demonstrated to interact physically and genetically with ELONGATED HYPOCOTYL5 (HY5) which encodes a bZIP-type transcription factor that functions downstream of multiple photoreceptors to promote photomorphogenesis (Zhang et al., 2014). This dual interaction allows a feedback mechanism for linking the light- and Cu-responsive gene networks and confirms that the tight spatio-temporal regulation of Cu homeostasis is part of the integral cellular circadian system (**Figure 1**; Andrés-Colás et al., 2010; Zhang et al., 2014).

### Copper Deprivation Induces a Switch from Optimal to “Economy” Mode

To face Cu scarcity in soils, plants have developed two strategies. The first one consists of increasing Cu acquisition by activating high-affinity Cu transporters such as members of the COPT/Ctr protein family as well as the expression of specific Cu chaperones (CCH; **Figure 1**; Himelblau et al., 1998; Abdel-Ghany and Pilon, 2008). The second strategy relies on the synthesis of non-Cu-containing proteins able to perform similar functions as cuproproteins. As example, the accumulation of ferric superoxide dismutase 1 (FSD1) can counterbalance the decrease in cytosolic and stromal Cu/Zn superoxide dismutases (respectively, CSD1 and CSD2) in the case of Cu deprivation (**Figure 1**; Abdel-Ghany and Pilon, 2008). This switch to “economy” mode is almost fully performed by miRNA mediation (**Figure 1**; Abdel-Ghany and Pilon, 2008). The Cu-responsive transcription factor SPL7 activates the transcription of transporters from the COPT/Ctr family and the production of miRNAs (denoted Cu-microRNAs) that target transcripts of “less-essential” cuproproteins in order to favor the allocation of Cu to plastocyanin, the most essential Cu protein in the chloroplast (Tapken et al., 2012). In *Arabidopsis*, the miRNA regulation involves several miRNAs (miR397, miR398, miR408, and miR857) which are predicted to target the transcripts of Cu proteins such as CSDs, Cox5b-1 a subunit of the cytochrome-c oxidase, plantacyanin and the laccase gene family (Abdel-Ghany and Pilon, 2008). In this mode, chloroplastic Cu-chaperone to Cu/Zn superoxide dismutases (CCS) and CSD2 are also downregulated, whereas the Cu-transporting ATPase PAA2, required for Cu delivery into the lumen, is stabilized and accumulates (Tapken et al., 2012).

## Copper Trafficking In Plants

During plant growth and development, ions are taken up by epidermal root cells, transferred to the center of the roots through the parenchyma and the endodermis and loaded in the xylem. This unidirectional transport of transition metals to the xylem is accomplished by different transporters. A coordinated activity of these transporters and other sequestrators/chelators is required for an adequate distribution of the minerals in all tissues at all stages of development.

### Absorption of Cu at the Root Site in Dicots

#### Cu Acquisition Involves Ferric Reductase Oxidases and COPT Proteins in *Arabidopsis thaliana*

**FIGURE 2:**
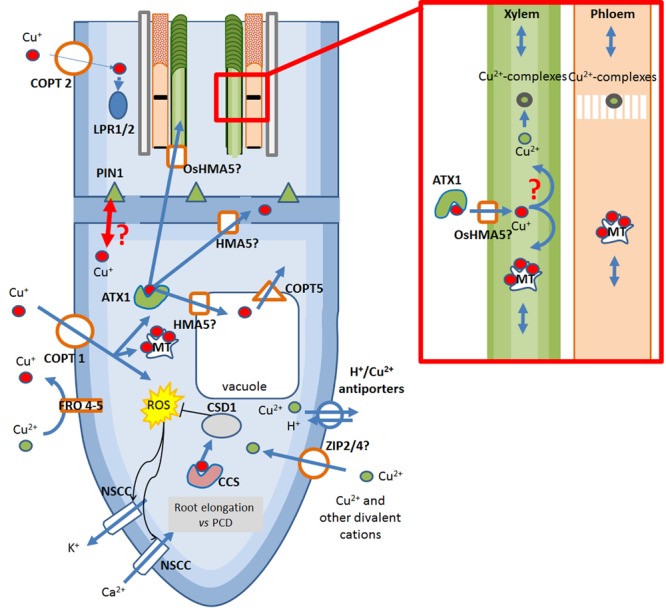
**Overview of the Cu-transport system occurring at the root tip of dicots.** Copper is taken up in the roots in its reduced form Cu^+^ by COPT proteins, highly selective Cu-transporters. Alternative, but still controversial, Cu uptake system may be non-selective ZIP proteins whereas Cu^2+^-eﬄux is mediated by H^+^/Cu^2+^ antiporters. In the cytosol, free Cu^+^ induces the generation of ROS thereby opening NSCCs, allowing the entry of Ca^2+^ and inducing root growth. Cu may further modulate root growth through interaction with auxin eﬄux carrier PIN1 and with the multi-copperoxidases LPR1/2. In case excessive Cu enters the roots, the massive generation of ROS activates also the eﬄux of K^+^ through NSCC, causing activation of Programmed Cell Death (PCD). ROS can be quenched by the acitivty of CSD1 which acquires Cu from CCS. To prevent ROS generarion, Cu^+^ is usually chelated by intracellular MTs or specific chaperones ATX1. Cu-transport to extracellular compartments in mediated by HMA5 proteins. In the phloem and in the xylem, Cu in transported in Cu(II)-complexes or Cu(I)-MT complexes. ATX1, antioxidant protein 1; COPT1/2, Cu transporter 1/2; CCS, Cu-chaperones to Cu/Zn superoxide dismutase; CSD1, cytosolic Cu/Zn superoxide dismutase; FRO 4/5, ferric reductase oxidase 4/5; LPR1/2, low phosphate response multi-copperoxidase 1/2; MT, metallothioneins; NSCC, non-selective cationic channels; OsHMA5/AtHMA5, *Oryza sativa* heavy metal ATPase 5/*Arabidopsis thaliana* heavy metal ATPase 5; PCD, programmed cell death; PIN1, pin-formed 1; ROS, reactive oxygen species; ZIP, Zrt- and Irt-like protein.

The mechanisms of Cu-uptake in plants have not been completely elucidated, however, a strong overlap between Fe-uptake and Cu-uptake mechanisms has been suggested (Ryan et al., 2013). In dicotyledons and non-graminaceous monocotyledons – referred to as strategy I plants – isotope fractionation analysis of Cu-uptake inside the roots indicates the preferential uptake of the light Cu isotope. Such observation has been proposed to result from reductive Cu-uptake mechanisms at the root surface, and thereby supports the idea that Cu(II) reduction to Cu(I) occurs at the root cell membrane of strategy I plants (**Figure 2**; Bernal et al., 2012; Jouvin et al., 2012; Ryan et al., 2013).

By analyzing the Cu(II)-chelate reductase activity in the roots of *Arabidopsis SPL7* mutants in comparison with wild type under different Cu availabilities, Bernal et al. (2012) found that Cu-deprivation induces high Cu(II)-chelate reductase activity at the root surface and that this activity is transcriptionally regulated by SPL7. Interestingly, these Cu(II)-chelate reductases are encoded by ferric reductase oxidases 4 and 5 (FRO4/5) which appear to mediate the specific reduction of cupric ions in particular at the root tips (Bernal et al., 2012).

In its reduced form, Cu is transported through the roots by Cu-uptake proteins from the previously mentioned Cu transporter COPT/Ctr-like protein family. Members of this family contain three transmembrane domains (TMDs), a His- and/or Met-rich N-terminus, a Mx_3_Mx_12_Gx_3_G signature motif embedded within TMD2 and TMD3, and except for COPT6, a cysteine/histidine Cu^+^-binding motif facing the cytoplasm at the C-terminus (Puig, 2014). Cu-limitation induces the expression of the three *COPT1/2/6* genes, but Cu-mediated regulation of *COPT3/5* has not been observed. A role of COPT4, which lack the classical Met residues essential for Cu(I) transport, remains to be elucidated (Puig, 2014). The expression profile of the COPT members also indicates that their regulation depends not only on the availability of cations but also on the tissue under consideration, on the developmental stage of the plant and on the environment in which the plants are growing, suggesting that Cu-acquisition is environmentally and developmentally regulated (Yuan et al., 2011).

Cu-acquisition from soil depends essentially on the protein COPT1. COPT1 is a plasma membrane-localized high-affinity Cu(I) transporter present in numerous organs and in *Arabidopsis* located more particularly at the root tips. Under Cu-limitations, the plant activates the expression of *COPT1* in a SPL7-dependent manner, allowing a highly efficient uptake of Cu from the culture medium (Sancenón et al., 2004). Copper entry into the cytosol is proposed to cause the generation of OH ⋅ in the cytosolic part of the cell via Fenton and Haber-Weiss reactions (Rodrigo-Moreno et al., 2013). The direct interaction of OH ⋅ with the cytosolic OH ⋅-binding site of non-selective cationic channels (NSCC), being plasma membrane ion channels, may then activate the influx of Ca^2+^ to allow root growth and thus increase the root prospection area for mineral acquisition. However, when excessive Cu is available, the massive entry of Cu(I) generates a burst of OH ⋅ radicals, leading to the activation of Ca^2+^-influx channels on one side and to the opening of K^+^ eﬄux channels on the other side, thereby activating a caspase-like driven programmed cell death (PCD) and inhibiting root elongation (Rodrigo-Moreno et al., 2013). In addition, excess Cu may disturb root elongation by preventing auxin redistribution through interaction with Pinformed1 (PIN1), an auxin eﬄux carrier responsible for acropetal auxin flow in the root stele (**Figure 2**; Yuan et al., 2013).

COPT2, another member of the COPT/Ctr-like family is localized in the plasma-membrane and shows an increased expression in response to Cu deprivation in a SPL7-dependent manner (Perea-García et al., 2013). *COPT2* is expressed in green tissues but also in the root differentiation zone, in the epidermis of the lateral roots and the root hairs but is absent from elongation and meristematic zones (Perea-García et al., 2013; Puig, 2014). Analysis of COPT2 activity in the reproductive organs of *A. thaliana* also disclosed expression in the stigma of young and older siliques, in the vasculature and the funiculus of older siliques as well as in anthers and pollen grains (Gayomba et al., 2013). Similarly to COPT1, COPT2 activity is altered by Cu-availability. However, Cu acquisition from soil by COPT2 seems limited and this transporter may only constitute a secondary pathway for root Cu incorporation (Puig, 2014). In addition to the GTAC motifs recognized by the SPL7 SBP-domain, COPT2 has in its promoter region a putative E-box involved in interacting with bHLH-type transcription factors, among which the Fe-responsive FIT protein, making this gene responsive to both Cu and Fe deficiencies (Colangelo and Guerinot, 2004; Perea-García et al., 2013). Interestingly, by mediating the delivery of Cu to the multi-copperoxidases LPR1 and LPR2 (low phosphate roots 1 and 2), involved in the root growth response to low phosphate, COPT2 may also play a role in phosphate sensing (**Figure 2**; Svistoonoff et al., 2007; Perea-García et al., 2013; Puig, 2014). COPT2 is thus believed to work at the intersection of many underlying processes between Cu and Fe homeostasis and phosphate metabolism, but the connection between the three processes are still poorly understood (Puig, 2014).

Next to this COPT/Ctr-like system of Cu movement, the expression of two transporters of the ZIP family, ZIP2 and ZIP4 able to mediate the transport of divalent cations, is modulated by Cu availability (Wintz et al., 2003; Del Pozo et al., 2010). These findings come from early gene expression profiles of *A. thaliana* cultivated under mineral deficiencies that have shown an induction of *ZIP2* and *ZIP4* expression in case of Cu limitation and a repression in Cu excess (Wintz et al., 2003). More recent studies, however, are partially contradictory since it was observed that Cu excess tends to mediate an up-regulation of *ZIP4* whereas *ZIP2* expression is induced by Cu deprivation (Yamasaki et al., 2009; Wu et al., 2015). This apparent contradiction in *ZIP4* regulation under Cu-deficiency may be explained by the different culture conditions and media used in the different studies. In addition, since *ZIP4* expression is regulated by Zn levels, a crosstalk may exist between Cu and Zn absorption (**Figure 2**). The role of the ZIP transporters in Cu(II) transport remains, however, controversial since none of these transporters rescue Cu uptake deficiency of Cu uptake deficient *ctr1/ctr3* yeast mutants (Milner et al., 2013).

#### Reducing Cu Toxicity by Chelation and Sequestration into the Vacuole

In plants, distinct systems involved in controlling the amount of free cytosolic ions have evolved. The first strategy is the down-regulation of the ion-specific uptake system, the second strategy relies on the removal of free ions and ion-ligand complexes into the vacuoles or on their eﬄux across plasma membranes into the cell wall (Krämer, 2005).

To avoid the generation of ROS, intracellular Cu must be chelated and delivered to its partner proteins by specific chaperones. In *Arabidopsis*, two specific chaperones [CCH and ATX1 (for Antioxidant Protein 1)] mediate the transfer of Cu to Cu-transporting ATPases (Shin et al., 2012). The study of *ATX1*-overexpressing lines under various conditions of Cu availability indicates a role of ATX1 in maintaining plant growth under Cu excess and mild Cu deficiency (Shin and Yeh, 2012; Shin et al., 2012). In particular, ATX1 appears to play a crucial action in the maintenance of the Cu homeostasis by chelating Cu through interaction with a conserved MxCxxC domain. Interestingly, Cu applied in excess also induces, in a dose-dependent manner, the specific expression of HMA5 (for Heavy metal ATPase 5), being a P_IB_-type ATPase. Regarding the strong interaction observed between *Arabidopsis* ATX1 and HMA5, ATX1 metallochaperones may play a fundamental role in the delivery Cu to HMA5 (Andrés-Colás et al., 2006; Del Pozo et al., 2010). However, since the exact localization of AtHMA5 is not elucidated, a role in Cu sequestration into the vacuole and/or in delivery to the apoplasm can be envisaged (**Figure 2**). In a recent study dealing with functional and biochemical characterization of cucumber Cu ATPases, two putative cucumber homologs of this protein, CsHMA5.1 and CsHMA5.2, were shown to be associated with the vacuolar membrane, contrary to the plasma membrane-localization of OsHMA5 in rice (Deng et al., 2013; Migocka et al., 2015). However, the differential regulation of *CsHMA5.2* but not *CsHMA5.1* in response to variations in Cu availability, in particular its high expression level under high Cu availability, suggests that CsHMA5.2 is responsible for an increased sequestration of Cu in case of Cu toxicity (Migocka et al., 2015). The plasma membrane-localized rice homolog OsHMA5, is in contrast suggested to be involved in metal eﬄux and not in metal sequestration into the vacuole, which highlights the species-dependent roles exerted by these HMA5 homologous and more specifically the different strategies that may exist between dicots and monocots (**Figure 2**).

Following root absorption of Cu and the sequestration of Cu into the vacuole, Cu ions can be exported from this storage compartment. The functional characterization of COPT5 in *Arabidopsis* has revealed that this protein is located in the tonoplast (Garcia-Molina et al., 2011; Klaumann et al., 2011). Yeast mutant complementation analyses further indicated that COPT5 is able to restore the growth phenotype of mutants unable to mediate Cu mobilization from Cu storage. It was postulated that Cu ions imported into root cells via COPT1 are stored in the vacuole until transport to the reproductive organs is required. In that case, vacuolar Cu is released via COPT5 and exported out of the cell to feed the needs of sink organs (**Figure 2**; Klaumann et al., 2011).

#### Copper Eﬄux at the Plasma Membrane May Enhance Cu Tolerance

Analysis of plasma membrane vesicles isolated from roots of plants of Cu sensitive and Cu-tolerant *Silene vulgaris* populations have shown that plants of Cu-tolerant populations can mediate Cu-eﬄux out of the cell via specific plasma membrane transporters (Van Hoof et al., 2001). Evidence for the existence of a Cu-transport system able to mediate Cu-eﬄux was obtained by Burzyński et al. (2005) while studying purified plasma membrane extracts from cucumber roots. Such eﬄux appears mediated by H^+^/Cu(II) antiporters, where the inward flux of H^+^ is compensated by an eﬄux of Cu^2+^. Although these antiporters appear to be saturable, this system of Cu export may contribute to the metal tolerance of the plant, notably by limiting the concentration of toxic metals inside the cell (**Figure 2**; Burzyński et al., 2005; Parrotta et al., 2015).

### Cu Translocation Along the Stem

#### Cu Is Transported in the Sap as Cu(II)-Chelates

In plants, Cu can be transported in the form of free Cu(I) or Cu(II) but it is generally done as Cu-complexes. Xylem loading is a key factor controlling the root-to-shoot translocation of minerals. However, Cu xylem loading in dicots is poorly understood and current knowledge comes largely from findings in rice. In rice knockout mutants of the P_1B_-type ATPase *OsHMA5*, the higher concentrations of Cu observed in roots in comparison to the wild type suggests an involvement of OsHMA5 in the translocation of this metal from the roots to the shoots (Deng et al., 2013). OsHMA5 is localized in the pericycle of mature roots and in the region of the vascular tissues in rice and transports Cu in the form of Cu(I) at both vegetative and reproductive stages (**Figure 2**; Deng et al., 2013).

**FIGURE 3:**
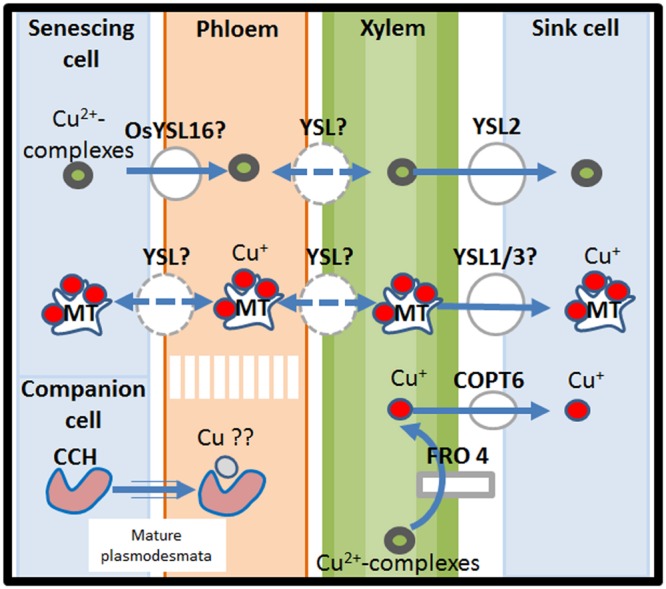
**A model of the long-distance system for Cu-transport.** Copper ions are transported in the vasculature in the form of Cu(I)-MT or Cu(II)-complexes. Prior to leaf cellular uptake, Cu^2+^ is reduced to Cu^+^ by FRO4 and enters the leaf cell by COPT6 proteins. Cu remobilization from senescing tissues is performed via YSL proteins. In particular YSL proteins similar to YSL16 in *O. sativa* are involved in the transport of Cu from Cu(II)-complexes from senescing organs to the phloem. A possible involvement of CCH in Cu remobilization from old to young tissues has also been envisaged. Remobilized-Cu is transferred to sink organs via YSL-proteins such as YSL2 for the transport of Cu(II)-complexes and probably YSL1/3 for Cu(I)-MT transport. CCH, Cu chaperone; COPT6, Cu transporter 6; FRO4, ferric reductase oxidase 4; MT, metallothioneins; YSL, Yellow Stripe Like.

In dicots possible re-oxidation of Cu(I) to Cu(II) has been suggested to occur, as observed in tomato, during Cu root-to-shoot translocation, to allow complexation with long-distance transporters (Ryan et al., 2013). Indeed strong evidence suggests that once in xylem sap, Cu is transported in the form of Cu(II) by specific metal chelators. Early investigations to find Cu ligands in xylem sap have focused on the ability of amino acids to form stable complexes with Cu. It arises from these analyses that free amino acids are able to mediate Cu transport (Liao et al., 2000; Irtelli et al., 2009). In addition, the non-proteinogenic amino acid nicotianamine (NA), whose synthesis is mediated by NA synthases (NASs), is an important player in mineral homeostasis (Hofmann, 2012). NA is synthesized by the enzymatic condensation of three amino-carboxylpropyl groups of three *S*-adenosyl-methionine molecules, by NAS (Curie et al., 2009). *In vitro*, NA can bind a wide variety of transition metals such as Mn, Fe, Co, Zn, Ni, and Cu (Curie et al., 2009). At the pH of xylem sap, NA and histidine exhibit the highest binding constants for Cu, which suggests that they may be the preferred ligands for the long-distance transport of this element, notably in xylem (Pich and Scholz, 1996). However, chelator speciation may occur depending of the availability of Cu, such as a higher involvement of NAs in case of Cu starvation than in case of Cu excess (Irtelli et al., 2009). In rice, Cu transport in the xylem sap may rely in contrast on 2′-deoxymugineic acid (DMA) as specific Cu-chelator, whereas NA and His may be the Cu-binding compounds in the phloem sap (Ando et al., 2012; **Figure 3**).

The transport of metal-complexes is carried out by members of the Yellow Stripe-Like (YSL) family, a subfamily of the OPT family of transporters (Lubkowitz, 2011; Zheng et al., 2012). OPTs are integral membrane proteins being protons-coupled symporters which bind to the metal chelator NA or the related set of mugineic acid family chelators (Chu et al., 2010). The best characterized OPT is the *Arabidopsis* transporter AtOPT3 that mediates a variety of roles, such as loading Fe into the phloem, facilitating Fe circulation from xylem to phloem, regulating shoot-to-root Fe translocation and is involved in iron redistribution from mature to developing tissues (Zhai et al., 2014).

The OPT family is divided in two clades, the Yellow Stripe-Like (YSL) and the oligopeptide transporter (PT), both involved in the transport of metal-chelates. The *A. thaliana* genome contains eight YSL genes, among which three (*AtYSL1, AtYSL2*, and *AtYSL3*) are expressed in the xylem parenchyma of leaves and are down-regulated by Fe deficiency (DiDonato et al., 2004; Waters et al., 2006; Chu et al., 2010). Localization of AtYSL1, AtYSL2, and AtYSL3 proteins *in planta* using green fluorescence techniques indicated that AtYSL1/2/3 are plasma membrane proteins (DiDonato et al., 2004; Chu et al., 2010). In particular, AtYSL2 and AtYSL3 localize jointly in the lateral plasma membrane rather than in the basal and apical extremities of the cell (DiDonato et al., 2004; Chu et al., 2010). Besides being involved in the maintenance of Fe homeostasis, the membrane protein AtYSL2 has been shown to be able to transport Cu-NA complexes (DiDonato et al., 2004). Overexpression of AtYSL3 in vegetative tissues induced higher accumulation of Cu in leaves whereas Zn and Fe were not affected, suggesting also a role of AtYSL3 in Cu-movement. However, yeast functional complementation with AtYSL1 and AtYSL3 both failed in showing evidence of Cu-NA uptake (**Figure 3**; Chu et al., 2010). In rice, Cu-chelate transport has been demonstrated for the phloem-localized transporter OsYSL16. It functions in loading the metal-complex in the phloem but is not involved in uptake from the medium. In particular, it serves to transfer Cu from senescing organs to the youngest plant regions and the seeds (Zheng et al., 2012). OsYSL16 also functions in iron homeostasis, confirming the link between Cu acquisition/redistribution and iron homeostasis (**Figure 3**; Lee et al., 2012). The regulation of YSL transporters upon variations of Cu-availability has been partially unveiled by analyzing a *siz1* mutant showing hypersensitivity to excess copper due to the incapacity to down-regulate the expression of *YSL1* and *YSL3* under excess levels of Cu (Chen et al., 2011). Double mutant analyses for *SIZ1/YSL3* and *SIZ1/YSL1* revealed that YSL3 plays a major role in Cu accumulation in shoots and that SIZ1 may participate in the regulation of the root to shoot translocation of Cu under Cu stress. Since SIZ1 is involved in the sumoylation process, the down-regulation of YSL1 and YSL3 under high copper availability in wild-type appears mediated by SIZ1-dependent sumoylation (Chen et al., 2011).

Once reaching the leaves, Cu(II) from Cu(II)-complexes is reduced to Cu(I) by the activity of FRO4 prior to leaf cellular uptake (**Figure 3**; Bernal et al., 2012; Ryan et al., 2013).

#### MTs, CCH, and COPT6 Mediate Cu Transport, Remobilization, and Redistribution from Senescing to Sink Organs

Metallothioneins (MTs) are cysteine-rich proteins able to coordinate Cu(I), Zn(II), and Cd(II) ions via their thiol groups (Guo et al., 2003; Wan et al., 2013). In *Arabidopsis, MT* genes are classified in four types (*MT1, MT2, MT3*, and *MT4*) and their accumulation changes in response to changes in Cu availability (Guo et al., 2003). *MT1a* and *MT2b* expression is mainly localized in the phloem in both leaves and roots and is induced by Cu, whereas *MT2a* and *MT3* are mainly expressed in mesophyll cells and are highly induced by increasing Cu levels in young tissues such as developing leaves or the root tip (**Figure 2**). MTs have been implicated in the mobilization of Cu from senescing organs. This has been suggested by the specific expression of *MT1a* and *MT2b* in vascular tissues; where *MT2b* is a housekeeping MT whereas *MT1a* may help the plant to deal with rapid variations in Cu-concentration (Guo et al., 2003). Whereas wild-type *A. thaliana* plants remobilize Cu from senescing leaves, *A. thaliana* quad MT-mutants *mt1a-2/mt2a-1/mt2b-1/mt3-1* do not. The quad-MT mutant also shows lower Cu-concentration in the seeds compared to the wild-type. Both these observations suggest that MTs are involved in Cu redistribution from senescing leaves to sink organs such as developing leaves or seeds (Benatti et al., 2014). Interestingly, a similar phenotype for Cu-accumulation was observed for the *A. thaliana ysl1/ysl3* mutant lacking YSL1/3 transporters, suggesting that MTs and YSL may interact (**Figure 3**; Waters et al., 2006; Chu et al., 2010).

It has been further observed that during leaf senescence, small Cu-binding proteins (CCH) accumulate in midrib vascular veins (class I vein) and the petiole of yellow-senescing leaves of *A. thaliana* (Mira et al., 2001). Immunohistochemical labeling revealed a particular accumulation of CCH in stem sieve elements but not in their companion cells (Mira et al., 2001). This, together with the presence of CCH in phloem exudates, suggests a role for CCH in long-distance Cu transport in vascular plants. In fact, CCH may be synthesized in companion cells and imported into the sieve elements through mature plasmodesmata where it mediates Cu-transport from senescing to sink organs (**Figure 3**; Mira et al., 2001; Sancenón et al., 2003; Andrés-Colás et al., 2006).

Finally, Jung et al. (2012) have characterized *A. thaliana* COPT6, a recently identified member of the Ctr/COPT family (Jung et al., 2012; Garcia-Molina et al., 2013). In contrast to COPT1 and COPT2, COPT6 lacks the Cys/His-Cu-binding motif at the C-terminus but retained its ability to mediate Cu transport (Jung et al., 2012). COPT6 is a plasma membrane-localized protein, with a primary role in Cu homeostasis in the above-ground organs, and more specifically in the appropriate distribution of Cu to leaves and seeds under limited Cu availability (Jung et al., 2012; Garcia-Molina et al., 2013). Interestingly, similarly to the Cu chaperone CCH, an involvement of COPT6 in Cu remobilization and redistribution from green tissues to reproductive organs has been proposed (**Figure 3**; Garcia-Molina et al., 2013).

### Intracellular Cu Transport

#### Cu Delivery to Chloroplast Involves Different P1B-type ATPases

Central components of Cu trafficking are small soluble proteins called Cu chaperones, able to mediate the delivery of Cu to its correct location (Blaby-Haas et al., 2014). In plants chloroplastic Cu must be transferred to the metallate plastocyanin, the plastid Cu/Zn superoxide dismutase (CSD2) and other proteins such as polyphenol oxidases. The correct allocation of Cu is required for photosynthesis and oxidative stress protection. In chloroplasts Cu transport is mediated by a system involving two Cu-transporting P_1B_-type ATPases [AtHMA6 (also known as PAA1 (P-type ATPases for *Arabidopsis*) and AtHMA8 (also known as PAA2)] respectively, located in the inner envelop and the thylakoid membrane (Shikanai et al., 2003; Abdel-Ghany et al., 2005; Catty et al., 2011; Blaby-Haas et al., 2014). These two proteins function in tandem; PAA1 imports cytosolic Cu to the stroma and PAA2 to the lumen (Blaby-Haas et al., 2014). Both PAA1 and PAA2 have highly conserved domains, a MxCxxC consensus metal-binding motif (HMBD) in the N-terminal region, a CPC transduction domain, a phosphorylation domain, a ATP binding domain and a phosphatase domain (Abdel-Ghany et al., 2005). *Arabidopsis* mutants for *PAA2* and *PAA1* have defects in photosynthesis but the phenotype is partially rescued by the addition of Cu to the growth medium, suggesting the presence of alternative low-affinity Cu transport routes that function independently from PAA1 and PAA2 (Abdel-Ghany et al., 2005). Evidence of this second transport system has been described in 2006, HMA1, another chloroplast envelop-located P_1B-_type ATPases, was found to be involved in providing Cu for photosynthesis. The *hma1* mutants contain less chloroplastic Cu, exhibit lower SOD activity and display a high photosensitivity under high-light conditions (Seigneurin-Berny et al., 2006), suggesting that HMA1 has a specific role in allowing the plant to grow under adverse light conditions (Seigneurin-Berny et al., 2006).

The delivery of Cu to Cu-transporting PAA1 is mediated by a plant specific Cu-chaperone, the plastid chaperone 1 (PCH1) encoded either by an alternative-splicing event of the *PAA1* pre-mRNA or by a separated locus, that preserve the N-terminal soluble portion of PAA1 and the Cu-binding motif MxCxxC. Relevant with the possibility of small folded proteins to be imported through the outer envelope, it has been speculated that PCH1 may bind Cu in the cytosol, pass through the outer envelope before delivering Cu to PAA1 (Blaby-Haas et al., 2014). Cu transport to PAA2 may in contrast involve stromal CCS devoted in delivering Cu^+^ to CSDs (in cytosol and in stroma) but which may also have evolved in parallel to interact with PAA2 in *Arabidopsis*, allowing the delivery of Cu^+^ to the essential cuproproteins PCs, and not solely to the non-essential CSD (Blaby-Haas et al., 2014). In case of high Cu availability, the chloroplast is able to regulate Cu transport into the thylakoid by the specific degradation of PAA2 via the chloroplast caseinolytic protease (Clp) system (Tapken et al., 2015a,b), indicating that PAA2 is directly involved in the homeostatic regulation of Cu in the chloroplast.

#### Cu Delivery to Mitochondria

Not much is known about the transport of Cu into plant mitochondria. Most knowledge results from comparison with yeast homologs. Balandin and Castresana (2002) found AtCOX17, an *Arabidopsis* protein which shares approximately 60, 33, and 51% identity with COX17 from rice, yeast and humans, respectively (Balandin and Castresana, 2002). AtCOX17 successfully complemented the respiratory deficiency of the Yeast COX17 null mutant indicating that AtCOX17 is a functional homolog of the yeast COX17, i.e., that it may mediate the delivery of Cu to mitochondria (Balandin and Castresana, 2002). Careful examination of the *A. thaliana* genome revealed that two genes encode similar yeast COX17 homologs, respectively, AtCOX17-1 and AtCOX17-2 which functions remain to be elucidated. However, since COX17 is a highly conserved protein, one can assume that the six conserved cysteine residues of Cox17 that participate in the ligation of Cu(I) in both yeast and mammalia perform a similar function in plants to allow Cu transfer from cytosol to the mitochondrial inter-membrane space (Palumaa et al., 2004; Giegé, 2007).

In yeast, SCO1 (Synthesis of cytochrome c oxidase 1) encodes a mitochondrial inner membrane protein necessary for the correct assembly of complex IV. SCO1 is a Cu-binding protein having the cytochrome c oxidase (COX) subunit 2 as direct interacting partner. In *A. thaliana*, two homologs of SCO1 have been identified and named, respectively, HCC1 (At3g08950) and HCC2 (At4g39740) (for Homologous of Cu Chaperone SCO; Steinebrunner et al., 2011). Although a high homology is reported for the two AtHCC, the CxxxC motif and one histidine residue which are essential for Cu ligation, are not conserved in HCC2, suggesting a distinct role of both proteins. In particular, HCC1 loss of function was shown to disrupt COX activities whereas HCC2 may rather indirectly act in redox regulation (Attallah et al., 2011; Steinebrunner et al., 2014). Finally, the presence of a COX11 homolog in the *Arabidopsis* genome, being potentially able to bind Cu(I) and to participate in the formation of the Cu_B_ metal center of the COX, suggests a role of this later at some terminal stage of cytochrome c oxidase synthesis, notably in inserting Cu into subunit I (Horng et al., 2004; Giegé, 2007).

In plants, multiple *Arabidopsis* FRO family members localize to organellar membranes, suggesting functional roles in subcellular compartments and supporting the idea that FROs might serve functions other than Fe/Cu reduction at the root surface (Jeong and Connolly, 2009). Histochemical staining of *FRO3-GUS* plants revealed that FRO3 is predominantly expressed in the vascular cylinder of roots and subcellular prediction of this reductase indicates a mitochondrial localization. Since Cu limitation induces the expression of this protein in roots and shoots, this yet uncharacterized reductase may be involved in copper homeostasis in mitochondria (Mukherjee et al., 2006; Jain and Connolly, 2013; Jain et al., 2014).

#### Copper Transport through the Secretory Pathway: Focus on Ethylene Receptors

Ethylene is a phytohormone controlling different aspects of plant development such as cell growth and cell shape, thickening of the stem, fruit ripening, and plant responses to biotic and abiotic stresses (Bleecker and Kende, 2000). Ethylene is perceived by plants through interaction with its proteinaceous receptor, a homodimer of Ethylene Receptor 1 (ETR1; Rodriguez et al., 1999). This receptor is made of (1) several TMDs forming an electron-rich hydrophobic pocket involved in Cu- and ethylene-binding and (2) a C-terminal domain located in the cytosol necessary for signal transduction (Rodriguez et al., 1999). Copper delivery to ethylene receptors is done by Cu-transporting proteins similar to the CPx class of P-type ATPases such as CCC2 protein from *Saccharomyces cerevisiae* able to mediate the transfer of Cu to extracytoplasmic compartments. This transfer implies that this transporter and the receptor share the same location during the biogenesis of the receptor (Binder et al., 2010). However, evidence of co-localization is lacking (Garcia-Molina et al., 2014b). In *Arabidopsis*, this class of P-type ATPase transporters includes the *response to antagonist 1* protein (RAN1, also known as AtHMA7) which has been shown to be essential for the biogenesis of ethylene receptors and crucial for normal growth and development (Binder et al., 2010). Copper acquisition by RAN1 may involve a system similar to Cu(I) transfer from ATX1 to apo-CCC2 in yeast, where Cu(I) induces the formation of an intermolecular metal-bridged complex made of the metallochaperone and the ATPase (Banci et al., 2006; Puig et al., 2007). In particular, AtATX1 interacts *in vivo* with the metal-binding domain of RAN1, whereas its homolog AtCCH, which has an extended plant-specific carboxy-terminal domain, does not (Puig et al., 2007). Recently, RAN1 has been shown to be induced by Cu supplementation in Cu-tolerant and Cu-sensitive ecotypes of *S. vulgaris*, suggesting a putative role of this protein in the global Cu homeostasis rather than in Cu tolerance mechanisms (Baloun et al., 2014).

#### Discovery of TaCT1, a Novel Cu Post-golgi Transporter Found in Common Wheat

Next to the two classical groups of Cu transporters, the Ctr/COPT Cu transporters and the P_1B-_type ATPases, Cu-transport in wheat involves a third class of proteins, the major facilitator superfamily (MFS)-type Cu transporter (Li H. et al., 2014). This protein, called TaCT1 (for *Triticum aestivum* Cu transporter 1), has a similar MxCxxC domain than the P_1B_-type ATPases. However, in this case this is located within the membrane of the post-Golgi compartment making it unable to interact with soluble ATX1 proteins (Li H. et al., 2014). In common wheat TaCT1 regulates Cu homeostasis and improves the tolerance to Cu stress (Li H. et al., 2014).

## Copper Effect on Cell Wall Components

The major sites of Cu-accumulation in plants are the chloroplast, the vacuole, the cytoplasm and the cell wall (Bernal et al., 2006; Burkhead et al., 2009). The cell wall is the primary site of contact for minerals and is characterized by a composite structure made of cellulose, hemicellulose, and proteins embedded in a matrix of pectins with variable degree of methylesterification. At the pH of the apoplast, unmethylated pectin residues are negatively charged and ionically interact with cations. The cell wall thus plays an important role in the heavy-metal response as ions sequestrator but its synthesis and composition can be severely affected in return (Parrotta et al., 2015).

**FIGURE 4:**
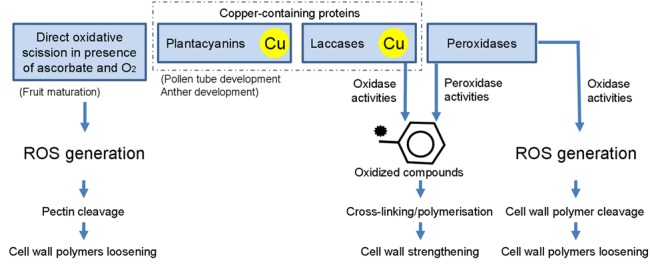
**Overview of some cell-wall loosening/strengthening mechanisms relying on the presence of Cu**.

The following part emphasizes the role of Cu on major cell wall proteins and cuproproteins and underlines how the fine tuning of the cell wall components, in particular the pectin-fraction, contributes to plant Cu-tolerance (**Figure 4**).

### Laccases Are Cu-Binding Proteins Involved in Lignin Polymerisation

The plant cell wall is a complex structure secreted by cells and forming a frame in which the cell develops. Young-growing cells develop a primary wall, mainly composed of cellulose and hemicellulose embedded in a matrix of pectins. In mature cells, a secondary wall made of cellulose fibrils with a higher degree of polymerization and lignin is produced and can particularly develop in specialized cells such as xylem vessels (Cosgrove, 2001). In plants phenolic compounds originate from the shikimate pathway, the products of which are either directed to protein synthesis or converted into secondary metabolites in phenylpropanoid metabolism. The phenylpropanoid pathway produces, among other compounds, the main lignin building blocks [guaiacyl (G), syringyl (S), and p-hydroxyphenyl (H) units]. After biosynthesis, the lignin units can be (1) transferred to the cell wall by specific ATP-binding cassette (ABC) transporters which pump the monolignol across the plasma membrane for free-radical–based oxidative polymerization or (2) imported into vacuoles by similar ABC transporters following a glycosylation step (Alejandro et al., 2012; Sibout and Höfte, 2012; Zhao et al., 2013; Dima et al., 2015). Heavy-metals are known to influence lignin deposition in a wide variety of plant species. In pepper (*Capsicum annuum L.*) seedlings Cu-excess triggers an increase in shikimate dehydrogenase (SKDH, EC 1.1.1.25), the enzyme that catalyzes the fourth step of the shikimate pathway and in peroxidase (EC 1.11.1.7) activities in both roots and hypocotyl and leads to an increased amount of phenolics and lignin (Diaz et al., 2001).

The monolignols are oxidized in the apoplast by members of the Cu-containing laccase and/or peroxidase families (**Figure 4**). Polymerization of the monolignols into lignin is a process during which oxidized phenols present in the lignifying zone enter the process of lignification by free-radical-based coupling (Zhao et al., 2013). In laccases, Cu ions are bound in several sites referred to as type I (“blue” copper proteins), type II (“non-blue” copper proteins), or type III (dimers) centers, depending on the environment of the metal ion and spectroscopic characteristics. The activity of the laccase family relies on the coordination of four Cu ions within the three spectroscopically distinct centers (types I, II, and III), allowing for the one-electron oxidation of a reducing substrate coupled to the four-electron reduction of oxygen to water (Sedbrook et al., 2002). *Arabidopsis* triple mutants for the laccase genes *lac4lac11lac7* display an extreme dwarf phenotype and almost completely lack lignin in their vascular elements. However, the Casparian strip, a lignin-based structure, is not affected in this triple mutant, probably because polymerisation of lignin in the endordermis is mediated by the concerted activities of peroxidases (at least PER64) and specific NADPH oxidase called Respiratory burst oxidase homologs (RBOHs) and not laccases (Lee et al., 2013). In roots, exposure to Cu in the range of 1–100 μM Cu causes an increase in ROS content, enhanced catalase, laccase, and peroxidase activities and stimulates the production of lignin, whereas root elongation is severely diminished (Lin et al., 2005; Wang et al., 2011; Liu et al., 2014).

In *Arabidopsis*, seven members of the laccase gene family are predicted targets for miR408 (LAC3, LAC12, and LAC13) miR397 (LAC2, LAC4, and LAC17) and miR857 (LAC7), 3 miRNAs that accumulate under Cu-limiting conditions. Interestingly, Cu deprivation induces the expression of these miRNAs and is negatively correlated with the accumulation of transcripts for laccases (Bernal et al., 2012). Since Cu availability also affects laccases which are not predicted to be targeted by miR408, miR397, and miR857, Abdel-Ghany et al. (2005) proposed that other small RNAs might regulate these laccase transcripts. However, the Cu-deficiency induced reduction in *LAC* transcripts may not or only partially contribute to overall Cu-economy in roots and in shoots (Bernal et al., 2012). Since miR398 and miR408 are also up-regulated in response to water deficit in *Medicago truncatula*, a link between the plant response to water deficit, Cu homeostasis and lignification has been proposed (Trindade et al., 2009).

Unlike the multicopper oxidase laccases, the amino acids residues acting as ligands for type I and type III copper are absent in the multicopper oxidase-related protein SKU5 (Sedbrook et al., 2002). However, since the two His residues that coordinate the single type II Cu are present, this protein is able to bind one Cu. Due to this difference in Cu-binding capacities, different enzymatic activities may be attributed to laccases and SKU5. In particular, since SKU5 lacks the type I and type III Cu ions necessary to perform electron transfer, it may work in tandem with a redox cofactor (Sedbrook et al., 2002). It is a ubiquitously expressed glycosylated GPI-anchored protein localized in both the cell wall and the plasma membrane, suggesting that it can be released from the GPI *in vivo*. *Sku5 Arabidopsis* mutants showed root waving and twisting, a lower root length as well as higher hypocotyl twisting in comparison to the wild type. Although clear functional characterization is still lacking, different models have been created to explain the function of SKU5. Among these, it was proposed that SKU5 has a role in modifying, either directly or indirectly, the linkages between structural cell wall components (Sedbrook et al., 2002). SKU5 may also act in modifying the properties of the middle lamella to allow cells to slide past each other during growth, or affect components of the signaling pathway in the cell wall (Sedbrook et al., 2002).

### Cu^2+^ Influences Class III Plant Peroxidases (POXs), Phenylalanine Ammonia-lyase (PAL) and Lignin Content

Secreted POXs (EC 1.11.1.7) can oxidize various substrates (phenolic compounds, lignin precursors, and auxin, etc) in the presence of H_2_O_2_, but also produce ROS such as H_2_O_2_, $O_{2}^{•–}$, or OH^•^ through their oxidative function (**Figure 4**; Cosio and Dunand, 2009; Hadži-Tašković Šukalović et al., 2010). POXs are heme-containing glycoproteins encoded by a multigenic family that contains three different classes of peroxidases. POXs react with compounds containing hydroxyl groups attached to an aromatic ring such as lignin or suberin precursors. Monomers are converted by POXs into unstable phenoxy radicals able to couple leading to the non-enzymatic polymerization of the monomers into lignin, respectively, suberin. In the cell wall, POXs can also oxidize feruloylated polysaccharides or structural proteins, leading to macromolecules of higher complexity, thereby conferring structural strength to the cell and the plant (Ishii, 1997; Hiraga et al., 2001; Hadži-Tašković Šukalović et al., 2010). Since peroxidases participate in a wide range of processes, display peroxidase and oxidase activities and are present in numerous isoforms with both conserved and variable sequences, it is difficult to assign a role to them (Cosio and Dunand, 2009). Studies have highlighted that Cu-treatments concomitantly affect both lignin metabolism and POXs activities. In radish, Cu-treatment inhibited root elongation but increased cationic and anionic POXs activities. Similarly, in soybean and sunflower Cu-treatment inhibits root elongation and branching and causes a decrease in biomass production (Chen et al., 2002; Lin et al., 2005; Jouili et al., 2008). This decrease is associated with an increase in the POX activity, a higher activity of laccases and an increased root lignin content (Lin et al., 2005). In lupine roots, Cu affects lignin metabolism right from the first steps of the synthesis of its precursors, as suggested by the enhanced activity of PAL under Cu excess (Chmielowska-Bak et al., 2008)

### The Cell Wall-related Plantacyanin Blue Cu Proteins are Target of Cu-Responsive miRNA

Plantacyanins belong to the phytocyanin family of blue Cu proteins with a Cu-fixating site made of four amino acids (2 His, 1 Cys, 1 Met/Gln/Leu). Plantacynanins locate in the cell wall, are around 10–22 kDa in size, are classified as type I copper proteins based on their spectroscopic and magnetic properties, and are thought to act in the transfer of electrons between a donor and an acceptor (Sakurai and Kataoka, 2007; Feng et al., 2013). The presence of Cu on the surface of the protein may facilitate their participation in ROS production (Dong et al., 2005). Different roles have been attributed to these proteins. During pollination, plantacyanin have a central role in pollen tube and anther development (**Figure 4**). In particular, the lily chemocyanin which was the first plantacyanin functionally characterized, is highly expressed in the stigma and style and induces pollen tube chemotropism (Kim et al., 2003). Next to this critical role in plant reproduction, plantacyanins are commonly induced under stress exposure (heavy-metals, low temperature, and high salinity), suggesting that they are involved in plant defense (Ma et al., 2011; Maunoury and Vaucheret, 2011; Feng et al., 2013). Transcript sequences of *Arabidopsis* plantacyanins exhibit strong complementarities with Cu-microRNAs (Andrés-Colás et al., 2010; Zhang et al., 2014). In particular, the gene *TaCLP1* which encodes a putative plantacyanin protein in wheat, is a target for miR408 (Feng et al., 2013). Copper supplementation in wheat and *Arabidopsis* induces the downregulation of miR408 and the concomitant upregulation of plantacyanin genes, thereby confirming the mediation exerted by the miRNA and the existence of a cross-talk between copper availability and plantacyanin transcript accumulation (Abdel-Ghany and Pilon, 2008; Feng et al., 2013). Interestingly, silencing of the plantacyanin gene *TaCLP1* in wheat induced a decreased stripe rust resistance, suggesting that TaCLP1is involved in the pathway of resistance to this pathogen. However, the detailed mechanism still needs to be investigated (Feng et al., 2013).

### Pectin Methyl Esterification Degree Controls Heavy Metal Tolerance

Plant cell walls are rich in polysaccharides able to bind divalent and trivalent metal cations through interaction with their functional groups –COOH, –OH, and –SH (Krzesłowska, 2010). In plants, these interactions are essentially with polysaccharides rich in carboxyl-groups such as homogalacturonans (HGAs). Following their synthesis in the Golgi, HGAs are secreted into the extracellular region were they follow specific modifications orchestrated by pectin methylesterases (PMEs, EC 3.1.1.11) and pectin acetylases, which finally affects their metal-binding capacities (Krzesłowska, 2010).

Metal accumulation in the apoplast has been mainly studied in plant species or populations showing remarkable capacities to survive on metalliferous soils. In the metal excluder *S. paradoxa* the root apoplasm of Cu-tolerant plants commonly accumulates less Cu than that of sensitive ones and this seems to be linked to a decrease in total pectins and an increased degree of pectin methylation (Colzi et al., 2011). A high degree of methyl-esterified pectins and thus a lower number of free pectic acid residues in the cell wall was similarly reported in Al-tolerant maize (Eticha et al., 2005). Exclusion of Cu by the metal-tolerant *S. paradoxa* appears thus related with a fine remodeling of the cell wall polysaccharides, at least in the roots. Since a higher pectin concentration correlates with a higher Cu accumulation in both the apoplast and the symplast, lowering the pectin concentration and increasing the level of high-methyl-esterified pectins in metal-resistant species may lead to a reduced absorption of Cu by the roots, thereby limiting its accumulation in the plant and leading to a higher tolerance (Colzi et al., 2012). In the most Cu-tolerant populations of *S. paradoxa*, Cu also influences root lignification, vessel differentiation and the production of mucilage (Colzi et al., 2012, 2015).

In fact pectin methyl-esterification appears as an essential factor in the modulation of the tolerance to a variety of metals, in plant metal excluder and in hyperaccumulators. In the root of the Cd-hyperaccumulating ecotype of *Sedum alfredii*, a higher degree of methylated pectins was reported in comparison with the non-hyperaccumulating ecotype. This was associated with a lower pectin methyl-esterase activity in the hyperaccumulating ecotype in presence and in absence of Cd and with a sixfold higher Cd concentration in xylem sap. Globally, it suggests that Cd is retained more tightly in the root cell wall of the non-hyperaccumulating ecotype due to more free pectic acid residues, thereby negatively affecting the loading of the metals into the xylem of this plant (Li T. et al., 2014).

### Ascorbate-induced Hydroxyl Radicals Cause an Oxidative Scission of Plant Cell Wall Polysaccharides

Besides the indispensable role of Cu in a number of redox proteins, free Cu may generate hydroxyl radicals susceptible to cause the non-enzymatic breakage of cell wall polymers, leading to cell wall loosening. Basic requirements for OH^•^-induced cell wall loosening are the presence of O_2_, a transition metal such as Cu^2+^ and an appropriate electron donor such as ascorbate, able to reduce apoplastic Cu^2+^ to Cu^+^ and O_2_ to H_2_O_2_ (Fry, 1998; Fry et al., 2002). Such generation of ascorbate-induced hydroxyl radicals may notably occur during fruit maturation, where the concerted action of ascorbate and Cu(II) generates hydroxyl radicals in the apoplast, thereby causing a non-enzymatic scission of arabinogalactan–pectin complexes with a high Gal content (**Figure 4**; Dumville and Fry, 2003). *In vivo* evidence of OH^•^ production in the apoplast and scission of specific cell wall polysaccharides has been described in elongating maize coleoptiles, radicles and endosperm caps of germinating cress seeds (Müller et al., 2009). Increasing the charge-to-mass ratio of de-esterified pectin appears in addition to increase its susceptibility to ascorbate-induced hydroxyl radical scission. Since de-esterification is catalyzed by PME, increasing PME activity may thus lead to a higher susceptibility of the cell wall to this non-enzymatic scission of pectic polysaccharides (Dumville and Fry, 2003).

## Conclusion

In the last years, the knowledge on the regulation of Cu uptake, transport, and allocation throughout the plant has increased significantly, and major Cu-depending and controlling processes are being progressively elucidated. However, much information is still lacking, particularly concerning the way copper is imported into mitochondria and how it reaches its molecular targets in this organelle. Although the broad transport process of Cu in the stem is being investigated, many details of these processes remain to be explained, notably concerning the nature of the transporters and chelators that allow Cu-circulation between mesophyll cells and the vasculature. It is not clear how these players evolve during the life cycle of the plant and how it evolved in the plant kingdom.

In general it appears that the regulation of Cu-uptake, transport and sequestration is intimately linked with other developmental and environmental regulatory pathways. The molecular actors in this integration are partially known, however, the way this integration influences plant phenotype requires further investigation. In fact, most advances in recent years have been made through the use of targeted analyses. The building of metabolic maps using high throughput techniques may provide additional valuable information regarding the whole integration of the signals that are modified by Cu-availability.

## Author Contributions

All authors listed, have made substantial, direct and intellectual contribution to the work, and approved it for publication.

## Conflict of Interest Statement

The authors declare that the research was conducted in the absence of any commercial or financial relationships that could be construed as a potential conflict of interest.
